# Time to consider sharing data extracted from trials included in systematic reviews

**DOI:** 10.1186/s13643-016-0361-y

**Published:** 2016-11-03

**Authors:** Luke Wolfenden, Jeremy Grimshaw, Christopher M. Williams, Sze Lin Yoong

**Affiliations:** 1 Hunter New England Population Health, Locked Bag 10 Longworth Ave, Wallsend, New South Wales 2289 Australia; 2 School of Medicine and Public Health, The University of Newcastle, Newcastle, 2289 Australia; 3 Ottawa Hospital Research Institute, Ottawa, ON K1N 6N5 Canada

**Keywords:** Systematic reviews, Review, Open data, Data sharing

## Abstract

**Background:**

While the debate regarding shared clinical trial data has shifted from whether such data should be shared to how this is best achieved, the sharing of data collected as part of systematic reviews has received little attention. In this commentary, we discuss the potential benefits of coordinated efforts to share data collected as part of systematic reviews.

**Main body:**

There are a number of potential benefits of systematic review data sharing. Shared information and data obtained as part of the systematic review process may reduce unnecessary duplication, reduce demand on trialist to service repeated requests from reviewers for data, and improve the quality and efficiency of future reviews. Sharing also facilitates research to improve clinical trial and systematic review methods and supports additional analyses to address secondary research questions. While concerns regarding appropriate use of data, costs, or the academic return for original review authors may impede more open access to information extracted as part of systematic reviews, many of these issues are being addressed, and infrastructure to enable greater access to such information is being developed.

**Conclusion:**

Embracing systems to enable more open access to systematic review data has considerable potential to maximise the benefits of research investment in undertaking systematic reviews.

## Background

The potential benefits of sharing clinical trial data have been well documented. A move toward a more open science of shared clinical data will enable replication of analyses, reduce waste, promote secondary analyses, and facilitate more efficient and robust evidence synthesis [1–3]. In 2011, the Cochrane collaboration first called for open access to data from all clinical trials [4], a view which has been broadly supported by researchers [5], international journals [6], and funding agencies [7]. While the debate regarding data sharing has shifted from whether clinical trial data should be shared to how this is best achieved, the sharing of data collected as part of systematic reviews has received little attention.

## The potential benefits of shared systematic review data

A variety of information obtained from published manuscripts, reports or other repositories, or following requests made directly to trial authors during the review process could be made more accessible. Information could include sample, effect size, and variability estimates from individual trials used in pooled analyses, information on trial methods or processes, or reviewer classification or assessment (i.e. risk of bias) of trials. Greater sharing of such information may offer a number of potential benefits. First, as is the completion of a clinical trial, undertaking quality systematic reviews is a considerable task and is estimated to take well-supported teams 12 months to complete [5]. Cochrane reviews, for example, require multidisciplinary expertise and resources to screen citations and extract data, to contact trial authors for additional information, to assess trial quality, and to analyse, interpret, and report review findings. Shared information and data obtained as part of the systematic review process may reduce unnecessary duplication, reduce demand on trialist to service repeated requests from reviewers for data, and improve the quality and efficiency of future reviews.

Second, data sharing may facilitate research to improve clinical trial and systematic review methods. Re-analysis of data from trials included in 146 meta-analyses including 1346 trials provided empirical evidence that inadequate allocation concealment or lack of blinding significantly increased estimates of treatment effects for subjective outcomes [8]. This finding was not significant when examining trials with objective outcomes. Further, analysis of data shared from trials by authors of the Cochrane Review of interventions to prevent obesity in childhood has been used to explore the use of a tool to classify research trials as pragmatic and explanatory [9]. The study identified that such study design characteristics may be important effect modifiers and recommended the tool be considered for use in systematic reviews.

Third, shared data and information extracted from systematic reviews can also been used to support additional analyses to address secondary research questions. Secondary analyses of existing review data may be particularly appealing for health policy makers and practitioners interested in quickly identifying effective interventions relevant to their context. For example, secondary analysis on trial data from a subset of studies included in a recently updated Cochrane review [10] was performed on behalf of health services to describe the potential effectiveness of interventions delivered by usual health service staff in reducing child exposure to tobacco smoke [11]. The findings were used to inform local health service practice regarding the type of support provided. Each of these examples of the benefits of shared systematic review data relied on agreement from review authors to release extracted trial data for re-analysis.

## Barriers to data sharing

Clinical trialists report a number of barriers to sharing clinical trial data, including concerns regarding appropriate use of data, costs associated with data sharing, or the ability of the trialist to have a fair opportunity to first publish research from the data set [1, 12, 13]. Such barriers are likely to similarly impede the sharing of data harvested through the process of a systematic review. Any errors incurred in the original trial data extraction process in systematic reviews would be perpetuated in secondary analyses of such shared data. The lack of standardisation on data extraction processes may result in increased time required by the review team to prepare information and data in a way that can be easily interpreted by those uninvolved in the extraction process. Furthermore, review teams may perceive requests for information and data extracted from reviews as unwanted competition that may challenge their opportunity to publish research manuscripts utilising such information, particularly when such use of data is not appropriately acknowledged.

## Moving forward: sharing of data extracted from trials during reviews

Currently, much of the information from individual trials obtained, extracted, and used in systematic reviews is not provided in published reports. A more open and coordinated approach to sharing review information may help capitalise on the potential benefits of sharing in a way that is considerate of the barriers and concerns of review stakeholders. Specifically, the development of infrastructure to centrally house and standardise data extracted on trials by reviewers and with data quality processes and criteria for data release may reduce the time reviewers are required to attend to data requests and may address concerns regarding appropriate use of data [2]. To this end, the Agency for Healthcare Quality and Research is funding a Systematic Review Data Repository (SRDR) being developed by the Evidence-based Practice Centre at Tufts Medical Centre [14]. The SRDR will act as a central archive and data extraction tool is freely accessible and enables researchers to incorporate previously deposited data into their reviews. Governance models and processes for enabling access and use of data have been included in the SRDR initiative to address some of the concerns regarding data sharing. Similarly, the Open Science Framework, developed by the not for profit Centre for Open Science as a means of improving collaboration in scientific research allows workflow and archiving of all research materials, including protocols and data sets, and provides user controls on release of information.

While sharing data can increase academic citation [15], systems to ensure appropriate acknowledgment of original review authors responsible for deposited information in any secondary analysis and methods to track the use and impact of such information may increase the academic incentive for sharing [1]. For clinical trials, the International Committee of Medical Journal Editors propose that, in order to be considered for publication in its member journals (including leading medical journals such as BMJ, The Journal of American Medical Association, and the Lancet), authors conducting secondary analysis using shared data must cite the original data source including the unique trial identifier to enable tracking of data use and invite collaboration by the study team depositing data [16]. Such provisions could similarly be extended to data collected via systematic reviews.

## Conclusions

The move toward more open system of data sharing is gaining momentum. A recent survey of reviewers affiliated with the Cochrane Collaborations Individual Participant Data Review Group, for example, found that 83 % supported sharing of the data collected as part of systematic reviews in a central repository [17]. While a number of barriers to data sharing exist, for clinical trials, these are being addressed, including in a recent report by the Institute of Medicine (IOM), journal editors, and funding agencies [2, 3]. The experiences of the SRDR and the recommendations of the IOM committee and others would serve a sensible point of reference for institutions publishing and supporting the production of systematic reviews who are interested in increasing the availability of data extracted as part of systematic reviews.

## Abbreviations

IOM: Institute of Medicine
SRDR: Systematic Review Data Repository

## References

[1] Ross SJ, Krumholz HM (2013). Ushering in a new era of open science through data sharing. JAMA.

[2] Institute of Medicine Committee on Strategies for Responsible Sharing of Clinical Trial Data (2015). Sharing clinical trial data: maximizing benefits, minimizing risks.

[3] Mello MM, Francer JK, Wilenzick M, Teden P, Bierer BE, Barnes M (2013). Preparing for responsible sharing of clinical trial data. N Engl J Med.

[4] Gøtzsche PC. We need access to all data from all clinical trials [editorial]. Cochrane Database Syst Rev. 2011;(10). doi:10.1002/14651858.ED000035. 10.1002/14651858.ED000035 PMC10846431 22161455

[5] Wolfe N, Gøtzsche PC, Bero L (2013). Strategies for obtaining unpublished drug trial data: a qualitative interview study. Syst Rev.

[6] Groves T (2010). BMJ policy on data sharing. BMJ.

[7] National Institutes of Health (NIH). Final NIH statement on sharing research data. 2003. http://grants.nih.gov/grants/guide/notice-files/NOT-OD-03-032.html. Accessed 4 June 2015.

[8] Schulz KF, Chalmers I, Hayes RJ, Altman DG (1995). Empirical evidence of bias. Dimensions of methodological quality associated with estimates of treatment effects in controlled trials. JAMA.

[9] Yoong SL, Wolfenden L, Clinton-McHarg T, Waters E, Pettman TL, Steele E, Wiggers J (2014). Exploring the impact of pragmatic and explanatory study designs on outcomes of systematic reviews of public health interventions: a case study. J Public Health.

[10] Baxi R, Sharma M, Roseby R (2014). Family and carer smoking control programmes for reducing children's exposure to environmental tobacco smoke. Cochrane Database Syst Rev.

[11] Daly J, Mackenzie L, Freund M, Wolfenden L, Roseby R, Wiggers J (2016). Interventions by health care professionals who provide routine child health care to reduce tobacco smoke exposure in children: a review and meta-analysis. JAMA Pediatr.

[12] Rathi V, Dzara K, Gross CP, Hrynaszkiewicz I, Joffe S, Krumholz HM, Strait KM (2012). Sharing of clinical trial data among trialist: a cross sectional survey. BMJ.

[13] Krumholz HM, Peterson ED (2014). Open access to clinical trials data. JAMA.

[14] Ip S, Hadar N, Keefe S, Parkin C, Iovin R, Balk EM, Lau J (2012). A web-based archive of systematic review data. Syst Rev.

[15] Piwowar HA, Day RS, Fridsma DB (2007). Sharing detailed data is associated with increased citation rate. PLoS One.

[16] Taichman DB, Backus J, Bauchner H, de Leeuw PW, Drazen JM, et al. Sharing clinical trial data: a proposal from the International Committee of Medical Journal Editors. Ann Intern Med. 2016. doi:10.7326/M15-2928. 10.7326/M15-2928 26792258

[17] Smith CT, Dwan K, Altman DG, Clarke M, Riley R, Williamson PR (2014). Sharing individual participant data from clinical trials: an opinion survey regarding the establishment of a central repository. PLoS One.

